# Protection, preparation or processing support for medical students’ emotional challenges in work-based learning—a grounded theory

**DOI:** 10.1007/s10459-026-10512-4

**Published:** 2026-02-13

**Authors:** Linda Barman, Maria Weurlander, Henrik Lindqvist, Robert Thornberg, Annika Wernerson

**Affiliations:** 1 Department of Education, Stockholm University, Stockholm, Sweden; 2 Department of Clinical Science, Intervention and Technology, Karolinska Institutet, Stockholm, Sweden; 3 Department of Behavioural Sciences and Learning, Linköping University, Linköping, Sweden

**Keywords:** Clinical supervision, Emotions, Work-based learning, Clinical practice, Medical students

## Abstract

Research shows that students experience emotional challenges during work-based learning that may affect their professional becoming and well-being, but they seldom feel supported by clinical supervisors in coping with or learning from these situations. The aim of this study was to explore clinical supervisors’ role in supporting medical students’ experiences of emotional challenges in work-based learning. We conducted a constructivist grounded theory analysis based on interviews with 20 physicians from different medical specialties, including residents working in Swedish healthcare who had experiences of supervising medical students. The findings illuminate how supervisors use different strategies in regard to students’ experiences of emotional challenges. As constraints in healthcare make it difficult to find room for reflective conversations, support is often provided on an ad hoc basis. Not all supervisors considered themselves to be responsible for providing students with emotional support, believing instead that such activities should be scheduled into the curriculum offered by universities. Our findings suggest that, despite evidence that medical students hide emotional affect, a major part of the responsibility to signal need and be open for emotional support still rests on students. Clinical supervisors can contribute to a permissive climate, allowing for emotional reflections; and supervisor training can foster discussions on the types of situations medical students may experience as emotionally challenging.

**Supplementary Information:**

The online version contains supplementary material available at https://doi.org/10.1007/s10459-026-10512-4.

## Introduction

Medical students experience several emotionally challenging situations during work-based learning, and if these situations go unnoticed, they may negatively impact their learning and well-being (Fosnot et al., 2022; Steiner-Hofbauer & Holzinger, 2020). These challenges include encountering severely ill or vulnerable patients, but also feeling insufficient, and experiencing unprofessional behaviour among healthcare staff (de Vries-Erich et al., 2016; Weurlander et al., 2018, 2019). Studies report on insufficient support from clinical supervisors (CS) in this regard (Weurlander et al., 2018); and yet, research emphasises the importance for students to learn from and manage emotional challenges (Wald et al., 2019). Despite this, little is known about clinical supervisors’ recognition of their responsibilities and strategies for supporting medical students’ emotional challenges during clerkship.

The dual role of being healthcare provider and a supervisor in clinical settings can be challenging. Still, clinical supervisors are significant for medical students’ learning; for example, in creating a safe learning environment and in supporting their emotional growth (Dornan et al., 2015; Pront et al., 2016). With regards to helping students navigate emotionally challenging situations, supervisors may be unaware that students are emotionally affected, since students may be reluctant to share such experiences with them (Weurlander et al., 2019). Instead, during clerkship students seem to adopt a front stage character, committing to the role of being a future doctor who meets the expectations of healthcare professionals and others (de Vries-Erich et al., 2016). Moreover, the clinical culture may not be perceived as permissive when it comes to expressing emotions or negative feelings (Barman et al., 2023; Jeung et al., 2018; Weurlander et al., 2019). If students experience that the clinical culture is defined by detachment (Weurlander et al., 2019) and hardness (Barman et al., 2023) they can be reluctant to disclose emotional experiences. Thus, it may be difficult for clinical supervisors to recognise individual signs and acknowledge situations where students are emotionally challenged and distressed.

In addition, supervisors have different understandings of what their role entails; for example, is it limited to demonstrating clinical procedures or does it also include stimulating students’ growth (Stenfors-Hayes et al., 2011)? Furthermore, they are prepared to varying extents for their CS mission (Pålsson et al., 2023), and may perceive only limited opportunities to meet students’ learning needs (Milos Nymberg & Jakobsson, 2022). Supervisors are expected not only to teach practical skills and clinical reasoning, but also to act as role models, support students’ identity formation as doctors-to-be (Cruess et al., 2014; Wald et al., 2015), and foster the development of students’ empathy (Pohontsch et al., 2024). The literature suggests several ways for how to ensure effective clinical supervision, such as establishing relational trust and an open climate, facilitating reflective practice and undertaking supervisor training (Martin et al., 2014; Rothwell et al., 2021). Still, little is known about how supervisors go about supporting students’ emotional challenges and whether they recognise situations that students may find demanding. Aiming to explore the role of clinical supervisors in supporting medical students’ experiences of emotional challenges during work-based learning, we posed the following research question: *How*,* according to their experiences*,* do clinical supervisors support medical students in navigating emotional challenges during work-based learning?*

Clinical supervision is defined here as ‘the provision of guidance and feedback on matters of personal, professional and educational development in the context of a trainee’s experience of providing safe and appropriate patient care’ (Kilminster et al., 2007, p. 2). Such definition includes a wide range of responsibilities. However, since roles are constructed and re-constructed in social interaction processes (Charmaz, 2014), we recognise contextual variations and, accordingly, the different conditions for providing student support during the day-to-day clinical rotations.

### Emotions in medical education

Emotions are defined and researched in several ways, including as individual psychological processes that contrast cognition, or in terms of their function in society (Turner, 2009). Similar to research on learning and related phenomena in medical education, views on the nature of emotions span from individual aspects to social constructions. Research on emotions emphasises: (1) emotions as individual bodily states, (2) emotions as competence attached to skills and abilities, or (3) the sociocultural function that emotions serve in social exchange (McNaughton, 2013). Furthermore, emotions are connected to motivation, behaviour, and memory (LeBlanc et al., 2015). Students’ emotions can influence their learning and be experienced as either positive or negative states (McConnell & Eva, 2012). In this study, emotions are recognised as individual, subjective, and as a natural part of human life and learning. Emotional challenges may be experienced as positive and supportive of learning, but here we are concerned with emotions evoked by situations in which students feel negatively affected and even distressed. Such feelings vary in magnitude and may result in learning. However, studies also suggest that if students are left to manage these negative and stressful emotions on their own, the consequence may be a ‘non-learning’ experience, where affectual details of an event may remain in memory but the contextual factors are never processed or understood (Kensinger, 2009; Lindström et al., 2011).

## Method

The current study was conducted with a constructivist grounded theory (CGT) methodology (Charmaz, 2014) in which clinically active physicians who were also responsible for supervising medical students were interviewed. We chose this approach because CGT is well-suited for the exploratory investigation of understudied practices and phenomena. It is designed to explore and conceptualise social processes, actions, interactions, and meanings (Charmaz, 2014).

### Study context

In Sweden, supervision of medical students is formally conducted by junior doctors during their specialty training (residents), and by specialists, including senior consultants. Informally, supervision is sometimes provided by interns who have not yet obtained their medical licence. In the current study, we focus on physicians’ supervision (including residents) of medical students in in- and outpatient settings. Before recent reconstructions of the medical programme, this was largely provided during the last three years of the five-year programme. Depending on their speciality (e.g., surgery or paediatrics), supervisors meet students who are more or less familiar with clinical practice. Parallel to clinical practice, students participate in seminars and theoretical sessions provided by clinical teachers and university faculty. The doctors supervise individuals or groups of students, and meet the same student(s) on one day, or more or less continuously for one to two weeks. The supervisors’ primary responsibility is most often to provide medical care, and their involvement in the medical programme varies. Consequently, they are more or less familiar with the structure and requirements of the medical programme; for example, with intended learning outcomes and individual student needs. Part of work-based learning may be placements in interprofessional learning (IPL) wards, where supervisors have dedicated time to focus on student learning. Several initiatives have been implemented to strengthen work-based learning in Sweden, including integrating recurrent clinical placements from early semesters. However, since the participants’ supervisory experiences ranged between 2 and 40 years (or more), their experiences did not reflect recent reorganisations of the medical programme, or policy changes regarding work-based medical education on requirements for supervisor training (Swedish National Board of Health and Welfare, 2023).

### Participants

We utilised open sampling in the initial recruitment of supervisors (Hallberg, 2006), which involved purposively selecting participants to ensure a broad range of experiences and descriptions in the data. Accordingly, supervisors were recruited with the explicit aim of capturing diverse experiences across various clinical settings, as well as variation in participants’ age and specialties. The participant physicians worked at hospitals or clinics in different cities in Sweden. While the majority reported having had some form of training regarding clinical supervision, such as courses and/or workshops and collegial seminars, several informants were unsure and did not remember details of their participation in such training. By employing an open sampling procedure, the study sought to maximise heterogeneity in the sample during the early phase of data generation, enhancing the richness and depth of insights into supervisory practices within different clinical contexts, as recommended by Hallberg (2006). As the iterative interplay between data gathering and analysis led to the development of tentative categories, open sampling was then succeeded by theoretical sampling. CGT theoretical sampling means that new data help expand, revise, and explicate the tentative categories (Charmaz, 2014; Kennedy & Lingard, 2006). At this point, we invited participants who supervised students in the later stages of their studies, and sought to include data on other kinds of emotional challenging situations that may arise, increasing the possibilities for other CS action and CS–student interactions. Therefore, additional participant recruitment was based on CS specialty and workplace (e.g., a closed psychiatric ward and a surgical specialty in a private clinic).

In total, we conducted 20 semi-structured interviews with supervisors (12 women and 8 men) including residents and senior consultants. Participants were interviewed on-site (11) or online (9) due to practical geographical reasons. The informants were unable to recall the exact year they first began supervising medical students in work-based learning, and several indicated that they had undertaken such supervision during their own internships. Given the uncertainty of these data, we present an aggregated overview with a valid accuracy in terms of years (Table 1).

**Table 1:** Participants’ supervisory experience

Experience as a supervisor (years)	Number of participants
< 3	2
3–14	9
15–30	6
30+	3

**Table 2:** Participant supervisors’ clinical expertise, as related to current supervision

Supervisors’ specialties (including residents)	Fictitious name, participant supervisors, and clinical settings
Anaesthetics	Stephen, Carl, Nicole (hospital)
Psychiatry	Katerina, Svetlana, George, Rob (private clinic, hospital, rehabilitation centre)
Cardiology	Sonja, Peter, Sandra (hospital)
Paediatrics	Monika, Karen (hospital)
General practice	Anders, Johanna (primary care centre)
Internal medicine	Lise, Janet (hospital)
Neurology	Anna (hospital)
Urology	Sven (private clinic and hospital)
Palliative Medicine	Vivianne (Advanced Medical Home Care)
Orthopaedic Surgery	David (hospital, IPL ward)

In the presentation of findings, fictious names are used (see Table 2).

We present an overview of collective background data for several reasons. First, most of the participants had previously, or were currently, supervising while working at several different health units/providers in Sweden, and some had experiences from different parts of the world. Also, several supervisors had more than one specialty training (either in full or part) that was relevant for their supervisor experiences. Hence, their experiences were not reduced to their current specialty, main workplace, and years of formally being a supervisor.

### Data generation and analysis

Interviews were primarily conducted by the first author (17), although a few (3) were conducted by the second author, enabling shared reflection and the refinement of questions. Initially, an interview guide was developed but consistent with grounded theory, additional questions were added based on the ongoing analysis (Charmaz, 2014; Kennedy & Lingard, 2006). The interviews, all in Swedish, aimed to capture narratives about concrete situations in which the supervisors perceived that students had become emotionally challenged. In addition, the questions included their thoughts about clinical supervision and their conditions for providing student support. The analysis and additional recruitment of participants and interviews were conducted in parallel until theoretical saturation was deemed to have been reached. According to Charmaz (2014), theoretical saturation refers to “the point at which gathering more data about a theoretical category reveals no new properties nor yields any further theoretical insights about the emerging grounded theory” (p. 345). Given that inquiries and knowledge are always provisional, fallible, and constantly open to revision, this decision was inevitably based on our subjective interpretation (Thornberg et al., 2023) that sufficient data had been gathered (Charmaz & Thornberg, 2021). For this reason, Dey (1999) preferred the term ‘theoretical sufficiency’ over ‘theoretical saturation’. In other words, we concluded data generation when we judged that enough data had been collected to fully develop our categories and construct our grounded theory.

The first author conducted the analysis, which was discussed in depth with the second author, and, on several occasions, with all authors. The empirical materials were transcribed verbatim and analysed iteratively according to constructivist grounded theory method using open and focused coding centred on meanings and actions, and through constant comparison and memos (Charmaz, 2014). The initial coding process resulted in four focused categories capturing supervisors’ actions and strategies, before theoretical coding to identify relations between categories and sub-categories. The theoretical coding included analytical questions pertinent to our research aim, such as when, by whom, how, and why, which resulted in a process or ‘timeline’ for when different support strategies were enacted. During the parallel focused and theoretical coding, two more main focused codes were identified (curricular activities not performed by CS) that revealed the boundaries of the supervisory role.

### Reflexivity statement

The main part of this study was conducted by the first (LB) and second (MW) authors who had outsider perspectives in regard to supervising medical students in the clinical environment. None of the authors had prior relationships with the research participants, with one exception; AW (who did not conduct the interviews) had previously been part of the same medical education committee as one participant, albeit they were working at different hospital sites. Our multi-disciplinary research team were motivated to enhance knowledge on this topic because we had previously researched the emotional challenges among medical students, as well as among other professional students, for several years. Concurrent with CGT, data were seen as co-constructed between interviewer and participants. Our research team’s outside–inside perspective (regarding working and supervising in healthcare) were useful in the discussions on the analysis and of possible interpretations. AW is a physician with extensive experiences of work-based education in medical education. While her first-hand experiences were vital for understanding the context and the participants’ stories, the first author (LB) could keep her distance from the experiences as such, and during analysis focused on questions such as ‘what does this mean?’, and ‘what does this speak about?’. The first (LB) and second (MW) author, who conducted the interviews, had no leadership roles connected to the participants’ places of work or associated universities, and therefore no such power issues were identified.

## Findings

Several clinical supervisors in this study appeared to be unaware of typical situations in their clinical environment in which students were at risk of being emotionally challenged, or they found it difficult to recall instances where students had signalled such negative experiences. Supervisors who recognised students were emotionally challenged reported having used strategies and activities to *protect* or *prepare* students for potentially challenging situations. When supervisors suspected students were emotionally affected, they offered *processing support* or *passed on* information to colleagues or those with greater responsibility for medical education. Given that the supervisors’ main attention was on providing medical care, they tended to attend to students in *pre-* or *post*-challenging situations.

### Grounded theory of clinical supervisors’ role in supporting students’ emotional challenges

**Fig. 1:**
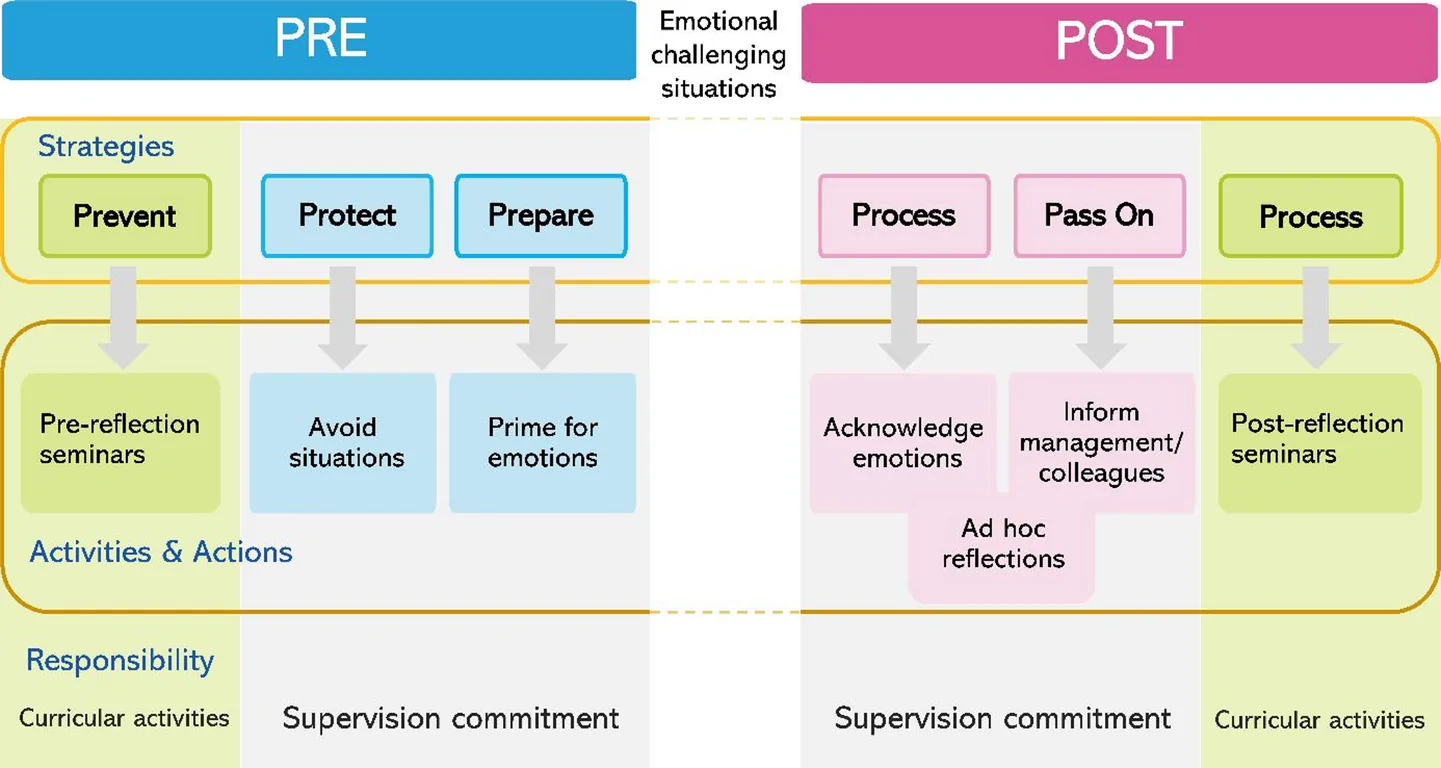
A grounded theory of clinical supervisors’ support and protection for students’ emotional challenges

First, we present our empirically grounded theory, which illuminates how several supervisors were committed to supporting or protecting students from emotional challenges, and highlights that some supervisors believed such support should be part of university-organised curricular activities. In healthcare, several situations involving patients made it difficult for supervisors to care for emotionally challenged students. Our model discloses supervisors’ role boundaries and their views on how support should be provided by colleagues or the university. The grounded theory highlights two main supports related to timing: (a) *pre-support*, including *preventing*, *protecting*, and *preparing;* and (b) *post*-*support*, which involves acknowledging the student’s emotional state and facilitating reflections by offering supervisory/curricular *processing support* or *passing on* information to others (Fig. 1).

Our presentation of supervisors’ role in supporting students’ emotional challenges starts with their strategies to either protect, prepare, offer processing support, or pass on information to others. Then, the boundaries of their responsibilities are made clear by presenting supervisors’ non-commitment, and in how some supervisors reported such support being taken care of by the medical programme (pre- or post-support), such as through scheduled prevention and post-seminars.

### Supervision commitment in supporting students’ emotional challenges

#### Protecting students from emotional challenging situations (pre-encounter)

One supervision strategy to support medical students was to protect them from emotional challenges by carefully organising their clinical placements. This approach aimed to ensure that students were not exposed to situations known to be emotionally challenging, especially during early semesters, when they were deemed unprepared to handle such challenges. For example, students were not expected to deliver difficult news to patients, such as a cancer diagnosis, or interact with children suffering from life-threating conditions. Instead, they were assigned work with ‘easier’ patients or asked to perform administrative tasks, as Monika explained:

Well, we choose patients from the ward that are supposed to be suitable, so we don’t let them take care of the most seriously ill, but it’s asthma and some infections. So, really not the hard cases. (Monika, paediatric care)

Supervisors acknowledged that protecting students from emotional challenges could be difficult due to the unpredictable nature of healthcare. In cases where patient encounters unexpectedly became emotionally difficult, supervisors emphasised the need to pay attention to student’s reactions and initiate a dialogue about the situation afterwards, if possible. Students, assumed to be in a vulnerable emotional state due to recent challenging situations, were supported by ensuring they were not left alone during the next patient encounters as a protective measure, as Karen elaborated:

If I’ve had a student during call and we’ve had a really tough case, that student might not want to be on their own the next time, but instead wish to walk alongside someone. So, you can just mention [to a colleague], ‘I saw you’re going with this student, could you just make sure they’re going with you?’ (Karen, paediatric care)

#### Preparing students for potentially emotionally challenging situations (pre)

Supervisors emphasised the individual differences among students and their varying needs for support. Younger students, particularly those without prior healthcare work experiences, often found their initial clinical encounters shocking. Recognising that these could be students’ first exposure to clinical practice, supervisors explicitly prepared them for situations known to be emotionally challenging. Such priming included non-verbal affirmations—such as seeking eye-contact or lightly placing a hand on the student’s arm or shoulder right before entering a patient meeting—and providing explicit guidance on how to manage stress. David explained:

I always say to those who participate in the operation theatre: ‘If you feel dizzy, it’s nothing to be ashamed of—everyone has been there—so sit down.’ It’s better to tell them in advance. ‘You don’t need to stand around and try to be some kind of hero. Everyone can react that way. It’s okay’. So, I’m usually careful to say that. (David, orthopaedics)

Preparatory actions were carried out just before potentially challenging patient encounters and were often informed by supervisors’ own emotional experiences in similar situations. Supervisors believed that even small gestures of acknowledgment could make a difference when time was limited. As illustrated in the above example, verbal or non-verbal pedagogical techniques could be employed effectively; even in time-constrained situations.

#### Processing support for students who experience emotional challenges (post)

Several supervisors noted that students occasionally needed support to process emotionally charged events, such as aggressive outbursts from patients or when dealing with social miseries or unexpected deaths. Acknowledging students’ emotional states required supervisors to be observant of those emotions. Supervisors who acknowledged these challenges believed that simply allowing students to talk about their experiences—even without offering solutions—was beneficial. Karen considered students in general to be wise, and that their life experiences should not be underestimated:

Most often it’s about getting it out in the open, and maybe [for the student] talking it through—and then it usually takes care of itself. (Karen, paediatric care)

Recognising that students may not willingly share emotional experiences, some supervisors employed deliberate strategies to facilitate reflections beyond the medical aspects of patient care. For example, Vivianne encouraged students who struggled after witnessing end-of-life care by emphasising how their work made a meaningful difference.

I usually ask if they found it difficult—and if they did, they generally say so. And then we talk about how this person [the patient] is doing and what we can do. I understand that this is hard, but there is a lot we can do that makes it better [for the patient]. (Vivianne, advanced medical homecare)

Often, at first, it is purely medical—they want to go through what happened: Why did that child arrive with this very low blood sugar? […] Why was she intubated in the ambulance […]? But then, when you’ve sort of gone through that […], you have to catch their attention and just say: ‘Now we’ve talked about this purely medical stuff, but I’m thinking, now you’ve seen a seriously ill child—does it awaken something in you? Was it difficult in any way?’ And then I feel that everyone answers when you really direct the question. And that’s what I think you have to do—you have to dare to ask that question and harbour the answer, and then you have to talk about it. (Karen, paediatric care)

Karen described a deliberate approach for encouraging emotional reflections. Despite these efforts, most supervisors admitted that reflective conversations in clinical environments were often spontaneous and unstructured, due to time constrains. While some supervisors attempted brief check-ins as they walked away from patient encounters, more in-depth reflections were typically postponed until the end of shifts or during scheduled sessions. George believed there were opportunities for students to talk to him, since he tried to spend time in the assistant medical office at the closed psychiatric ward where he worked:

It’s not that organised, but more spontaneous, when I have the time. (George, psychiatry)

Invitations to students to engage in reflection were ad-hoc and often dependent on the supervisor’s recognition of a situation constituting a possible emotional challenge. In extreme cases involving chaotic or traumatic events, debriefings with the entire healthcare team were occasionally arranged. However, such opportunities were rare. Vivianne recounted how her team processed an emotionally taxing case involving advanced homecare for a woman suffering from Hodgkinson’s lymphoma:

It was so terrible. We felt—everyone found it very hard to be there and everyone in the team was very grateful when she died. Because we—that suffering was hard to deal with. And once there was a student involved who became completely shaken.

Interviewer: did you talk about it?

Yes, of course we did, but it wasn’t just…[the student], everyone thought so (Vivianne, advanced medical homecare)

Several supervisors expressed a desire for students to participate more frequently in debriefings, but noted that such practices remained uncommon.

#### Passing on information (post-encounter)

Supervisors cited various reasons for passing on information about students who appeared emotionally affected after challenging events. Often constrained by time limitations, some supervisors asked colleagues to ‘check in’ with affected students later in the week or to have reflective conversations regarding their emotional state. Supervisors recognised that certain students required additional attention and support to cope with stress during work-based learning, but felt these responsibilities should involve the university or hospital management. Supervisors particularly relied on course leaders when addressing serious concerns—for instance, if they believed a student experienced discrimination or exhibited signs of mental health struggles. Course leaders or managers typically handled such cases by implementing strategies aimed at resolving issues and improving healthcare practices to prevent recurrence. However, Sonja noted one challenge:

In such situations, I usually try to have a dialogue with those concerned, but it’s not always that the student wants that, and then you feel a bit stuck. (Sonja, cardiology)

### Supervision boundaries and lack of commitment in supporting students’ emotional challenges

The supervisors were aware that situations during clinical practice could be experienced as emotionally stressful for students. Drawing from their own experiences as medical students, some supervisors believed that such situations were limited to specific sessions commonly known to evoke discomfort or disgust—for example, eye dissections or seeing an opened body during autopsies or surgeries. Beyond scenarios, which are generally perceived as emotional challenging in medical practice, such as seeing severely ill patients or dealing with death, several supervisors struggled to recall specific instances where a student required emotional support. Many believed that such occasions were rare. Peter explained:

It usually works fine. They are satisfied. You try to pick up if there is something they wish to share, but I haven’t got such signals yet. But I haven’t really dug any further, but maybe one should do that. (Peter, cardiology)

Some supervisors suggested that only a few students experienced emotional challenges, implying that those students might not be suited for the medical profession. They viewed it as falling outside their supervisory role to provide any emotional support beyond the guidance offered to all medical students. Rather, it was the university’s responsibility to arrange activities for students facing emotional difficulties. Lack of commitment to supporting students’ emotional challenges reflected disengagement because in such cases, the supervisors either did not consider emotional support part of their responsibilities, or assumed other support strategies were in place. Disengagement could also stem from limited opportunities or constraints within the clinical environment. Supervisors particularly highlighted stress and lack of time in emergency care—especially during night and weekend shifts when staffing was low—as barriers to prioritising students’ emotional needs. Scarcity of time and staff resources often forced supervisors to focus on more urgent matters, as Janet explained:

Everything depends in emergency care. How many patients happen to be hospitalised. If we are overcrowded so that there is a lot to do. […] Sometimes there are days when there is little time, and then, well, the supervision suffers from it. […] The worst is if you have patients who are really bad. Then it’s difficult to have an open dialogue; then I need to devote all my focus to that [the patient]. (Janet, internal medicine)

Several supervisors acknowledged that addressing students’ emotional challenges was rarely feasible ‘in the moment’. Sven and David explained why they were unable to attend to students who fainted or felt dizzy during surgeries:

I perform the surgery, so, someone else takes care of them—lifting the legs—and they wake up again. It’s not that dramatic if they don’t hurt themselves from the fall. (Sven, urology)

Well during surgery there are others who can bring a stool—people who don’t stand in the wound, so to say. (David, orthopaedics)

During surgery, other members of the healthcare team typically attended to students showing physical signs of stress. In other situations, however, supervisors reported that it was less clear whether students were emotionally affected and therefore more challenging to support. According to one supervisor, Monika, students could be emotionally affected by taking in ‘the whole picture’, including patients’ life situations and suffering. She stressed that since students were affected by different situations, it should ultimately be up to them to seek support if necessary. According to some supervisors, the boundary for clinical supervision was clear; support should be provided by the university if students needed guidance beyond learning medicine. Sonja, who had various roles, including course leader, reflected on her shifting responsibilities, and on the importance of distinguishing between listening empathetically and taking responsibility for escalating student concerns within the organisation:

Well, I think you have to consider how to draw the lines at all times. […] It’s easy to find yourself in situations where you are flattered by a confidence, but that is not my role. (Sonja, cardiology)

Policies and action plans existed for exceptional events requiring formal intervention by those responsible for medical education; however, not all supervisors were aware of the routines or the division of responsibilities between the healthcare organisation and the university. Some supervisors believed healthcare managers should take responsibility for supporting both students and staff during critical incidents. Peter shared his perspective:

Sometimes terrible things happen. It can even be a case of malpractice […]. I hope there is a structure for these things and that it is taken up by managers […]. According to me, managers should be active and handle situations like that. […] We had a tragic case this week—I don’t know what happened—but I hope it was acknowledged and that management took care of it so no one feels left alone. (Peter, cardiology)

#### Preventing students from experiencing emotional stress (pre-activities as part of the curriculum)

Supervisors reflected on their own previous experiences as medical students, in taking part in pre-reflection seminars designed to prevent emotional challenges. They believed it was a general idea in medical education that the curriculum should include pre-reflections. However, many were sceptical about whether discussions regarding emotionally difficult situations could truly prevent negative experiences from occurring. Anna explained:

But these experiences—as a doctor or medical student—and discussing difficult situations cannot be discussed preventively. They [students] have to experience and see it before it can even be discussed. […] So it’s not possible to prevent these situations, either—how it feels and how you function and what becomes difficult—you don’t see that until you start experiencing it. (Anna, neurology)

#### Reflections in the medical curriculum: processing emotional challenges (pre- and post-encounter)

Supervisors who recognised the importance of addressing students’ emotional states emphasised improving educational support in this regard but admitted they did not always know how to best provide this themselves. Scheduled reflection seminars organised by universities offered opportunities for medical students to discuss challenging experiences but did not necessarily involve clinical staff. Carl encouraged students to differentiate between maintaining ‘a cold mind’ during procedures while embracing ‘a warm heart’ at other times. He emphasised that concentrating on the medical procedure should not be confused with cynicism; rather, physicians must learn when it is appropriate to allow themselves to focus solely on their work versus allowing themselves space for emotional engagement and reflection:

At the heart of it all is getting to know yourself and your reactions, and practicing this ability, I think. Because it’s not given to us from the start. Practicing the ability to reflect on our limits and our reactions. (Carl, anaesthesiology)

One concern raised was that during organised seminars—including pre- and post-reflections on (potentially) challenging situations—students were often reluctant to share their feelings openly for fear of appearing insecure or unprofessional. Supervisors noted that these seminars frequently emphasised professional behaviour rather than acknowledging or processing emotional states.

### Barriers for recognising and supporting emotional challenges

Regardless of supervisors’ views on their responsibility, they all expressed that learning to cope with difficult situations was part of working in healthcare. Several supervisors emphasised the difficulty in getting students to share negative emotional experiences. They believed that this was partly because students chose to confide in friends or partners, or avoided appearing weak in front of supervisors or peers. Consequently, supervisors found it difficult to recognise when students needed emotional support, as Katerina and Sven shared:

My experience is that they don’t like to bring up these matters. […] I don’t think I’ve seen a single student who has happily addressed a somewhat negative situation. In fact, I almost think they’re trying to be extremely clever by, well, just biting the bullet and moving on. It’s actually a problem that they don’t address it. (Katerina, psychiatry)

According to supervisors, students seemed to believe that they were expected to keep emotions to themselves. Therefore, challenging events involving students were often brought to the supervisors’ attention by others, such as colleagues or patients.

## Discussion

Learning to handle one’s own and others’ emotions in challenging situations is an important part of student development in medical education (Dornan et al., 2015). Current and previous findings suggest that supervisors may find it challenging to recognise when students are emotionally distressed and in need of support. For example, students may conceal their emotional reactions, presenting themselves as meeting the expectations of a professional role (Barman et al., 2023; de Vries-Erich et al., 2016; Jeung et al., 2018). Aware of students’ reluctance to share their emotional affect, a few supervisors in the current study employed specific strategies to initiate conversations about patients’ medical conditions and treatments. Once students had exhausted their reflections about medical issues, supervisors then inquired specifically about the student’s emotional reactions. Acknowledging students’ immediate emotional reactions can minimise the risk of anxiety during work-based learning (Freeman et al., 2024; Jeung et al., 2018; Lindström et al., 2011; Pront et al., 2016). Also, given that long-term suppression of negative emotions is associated with various stress symptoms, and ultimately, burnout (Jeung et al., 2018), it seems crucial that supervisors implement strategies to elicit students’ emotional reflections and help them make sense of emotional challenges.

Some supervisors in the current study anticipated and prepared their students for potentially emotionally challenging situations. They subtly primed the students, reinforcing that emotional reactions are natural. Such priming can contribute to normalising emotional affect and are likely to mitigate students’ feelings of failure for reacting strongly. Exposing their feelings in front of patients and healthcare professionals may be shameful for students (Lindström et al., 2011). Making students aware of emotional reactions and their physical manifestations (e.g., feeling dizzy) immediately before challenging situations is likely to facilitate their willingness to signal whether they need support or to vent afterwards. However, supervisors can only prepare students for situations if they recognise potentially emotionally challenging circumstances. In this study, several supervisors struggled to identify such situations related to their own practices, beyond the well-known challenges of encountering death and severe illness. Many other situations in clinical work are identified as emotionally challenging and shameful for medical students, such as unprofessional behaviour and detachment among healthcare staff and the fear that insufficient knowledge could jeopardise patient safety (Lindström et al., 2011; Weurlander et al., 2018, 2019). Emotions seem to be increasingly recognised in medical education and physician practices (Dornan et al., 2015; McNaughton, 2013; Sargeant et al., 2008), and raising clinical supervisors’ awareness of situations that students may find challenging remains essential. We suggest that clinical supervisor training include discussions of various scenarios where students may feel emotionally challenged. Such training can potentially broaden their awareness of challenging situations and highlight how unrecognised and unsupported negative emotions can hinder learning and negatively impact well-being. Hopefully, this may lead to supervisors becoming more observant when it comes to students’ emotional states, and more inclined to invite reflection on emotions.

### Protecting or preventing students from experiencing emotional challenges

Medical students will inevitably encounter emotionally difficult situations as part of becoming doctors. Supervisors in the current study expressed different views on whether it is beneficial to *protect* students from such situations, since they need to learn how to cope with demanding experiences. They acknowledged that individual differences, maturity, and previous life experiences influence how well students are equipped to manage demanding situations. Students’ resilience, self-efficacy, and coping-strategies differ (Houpy et al., 2017; Lindqvist et al., 2021; Neumann et al., 2011) and supervisors may have or dedicate little time to establishing relationships with individual students that could inform them about who is ready to experience the realities of clinical life (Sellberg et al., 2022). Protecting students by organising clinical placement to avoid challenging situations may therefore be a valid approach early on, while students nearing the end of their studies may be better equipped to manage emotional challenges.

Several participating supervisors were familiar with, but also sceptical about, the impact of curricular activities in preventing students’ emotional stress. Our grounded theory clarifies the difference between supervisors’ situational in-clinic strategies aimed at *preparing* students for emotionally challenging situations (priming), and pre-organised seminars intended to *prevent* students from having negative emotional reactions in relation to clinical work. Supervisors in the current study questioned whether it is possible to prevent emotional affect. Instead, they emphasised the importance of students’ first-hand experiences of different clinical situations. We agree that it may not be possible, or even desirable, to prevent emotional reactions, considering the importance of learning to manage emotional stress and develop empathy (Dornan et al., 2015). Emotional challenges can serve as a catalyst for self-awareness, and students may even seek opportunities to understand their own reactions and needs in order to be better prepared for professional practice (Lönn et al., 2023). Reflections during pre-organised seminars can however serve other purposes, such as helping students recognise situations in which they can expect to react and, through this, contribute to demystifying emotions as a natural part of medical education and professional life. Different curricular activities supposedly acknowledging distress and promoting self-awareness are seen as one important part of students’ identity formation (Irby & Hamstra, 2016).

### If there is a time and place for reflections

Medical practice is filled with limited conditions for supervisors to attend to students, who consequently may be left to seek learning opportunities by themselves (Sellberg et al., 2022). Ad-hoc solutions for offering support seem to be common, which raises the question of how emotional support can be integrated into or offered in conjunction with clinical practices. As shown in the current study, acknowledging emotional experiences is not necessarily the same as the supervisor taking the time to sit down and ask the student about their feelings. If opportunities for processing support are truly limited by time-constraints, supervisors could acknowledge the presence of emotions in relation to challenging situations. Advising students to vent with peers or someone they trust, or to write down reflections for post-reflection seminars led by other teachers or mentors, are a few strategies (Freeman et al., 2024). Purposeful and focused reflections are suggested to be important for learning—if they are supported by someone who can facilitate an experience-near (authentic) relevant meaning-making process (Eraut, 1995). Post-reflection seminars provide students with time to deliberate, which may be important for making sense of residual emotional challenges and may contribute to counteracting empathy loss during clerkship (Mahoney et al., 2016; Neumann et al., 2011).

Acknowledging that healthcare is a team effort involving several health professionals, supervisors in the current study emphasised the importance of debriefings that include colleagues. Interventions that address emotional challenges are important for countering stress and burnout (West et al., 2016). Such actions often require more than individual supervisors offering students time for reflections, and resonate with approaches in organisational culture that recognise health professionals’ distress and are permissive of emotional processing. One concern is that students may feel like outsiders instead of being part of the healthcare team (Dornan et al., 2015; Weurlander et al., 2019), making it important for supervisors to invite students, also for debriefings, and help them feel part of the clinical environment. The CGT in this study suggests that invitations to reflect were ad-hoc and depended on supervisors’ recognition of students’ emotional distress.

According to the supervisors, reflective conversations during post-seminars often focused on professional behaviour, and left little opportunity for students to process their emotions. This raises a question as to whether there are opportunities for students to vent their emotions or if their experiences become ‘training material’ for mastering certain behaviours. Different views on how students develop professionalism are evident in medical education discourses that tend to emphasise physicians’ virtue, behaviour, or professional identity formation (Irby & Hamstra, 2016). The literature points to issues with supervisors being unfamiliar with the intended learning outcomes (Lindström et al., 2011; Sellberg et al., 2022), which may reflect an emphasis on behavioural approaches and formal assessable learning. Behavioural approaches tend to undervalue the intangible and informal learning involved in becoming a doctor, such as affect and emotions (Dornan et al., 2015). Learning to behave as a professional is important and should not be undervalued; however, there seems to be a gap in students’ opportunities to process their emotional reactions.

### Role boundaries—someone else’s responsibility

It is concerning that not all supervisors recognise the need to support students’ emotional challenges as part of their mission. Passing on information to someone who can act in a situation without compromising patient safety (e.g., during surgery) or in cases of severe student distress (e.g., mental health issues) to someone mandated to act seems reasonable if there are limited opportunities for sit-down reflections. However, it seems that several supervisors were unaware of who, or whether, student support was provided, which is concerning. The current study illuminates that supervisors have different views of their responsibility for students’ professional development as a whole, including emotional aspects. Dividing or sharing such responsibilities between the work-based and the university-based parts of education may have consequences for whether students are left with residual emotions or get support to cope with and learn from challenges. Many medical schools have significantly changed the way that clinical practice integrates with curricula over the past few decades, and demands for supervisors to adopt a holistic perspective are increasing (Dornan et al., 2015; Pront et al., 2016).

## Implications of the grounded theory

The theoretical model in this study illuminates the limited recognition of students’ emotional stress by supervisors during patient encounters and other emotionally demanding situations. Given the nature of healthcare and the competing demands of patient care, the findings call for a broader discussion on how student support responsibilities are shared within clinical teams. Furthermore, the model highlights support strategies pre- and post- challenging encounters, revealing the division of responsibility between clinical supervisors and curricular activities organised by university faculty. The CGT can inform improvements in medical education, particularly in relation to supervision training and curriculum development. Specifically, the model may assist educators in: (1) identifying how student emotions are acknowledged across the curriculum and where emotional support structures can be strengthened, especially in close proximity to work-based learning; (2) instigating discussions aimed at identifying supervisors’ conditions for addressing and supporting students’ emotional challenges, and on the collaboration between clinical- and university-based teachers regarding support responsibilities; (3) holding discussions on specific challenging situations, and in identifying realistic strategies to support students in learning how to navigate these situations; and (4) recognising students’ different needs regarding emotional challenges and support and in using the different strategies found here to better tailor support.

## Limitations and future directions

Duo to initial difficulties in recruiting clinical supervisors, several participants were recruited based on their involvement as mentors for groups of medical students. During mentor meetings (once or twice per semester), students reflect on experiences aimed at promoting professional development, which may have increased those supervisor’ awareness of students’ emotional challenges. The difficulty in recruiting participants may reflect an unawareness among physicians about their supervisor mission (Sellberg et al., 2022), or possibly a more widespread lack of commitment to supporting students’ emotional stress than is reflected among the interviewed participants. This study makes no claim to reveal the extent of how many or how often supervisors in general are involved in supporting students’ emotional affect, nor on how often students need such support. Instead, we call for more research in this regard. Another limitation is the uncertainty of each participant’s previous experiences or training regarding clinical supervision. However, consistent with our methodological grounding, our findings illuminate how the supervisor’s role may or may not support students’ emotional challenges, rather than how individual traits, experiences, or attitudes are associated with the enactment of different supervision strategies.

Additional research should evaluate strategies used in clinical practice for recognising and addressing students’ emotional challenges, and identify to what extent clinical supervisors recognise and provide support for students’ emotional challenges.

## Key findings and conclusions

Our findings indicate a need to raise clinical supervisors’ awareness of situations that students may find emotionally challenging, and increase knowledge about which strategies that can be useful for priming for emotions and inviting student reflections. Reflection on emotional demands in work-based learning appears to be ad-hoc, relying largely on supervisors’ acknowledgement of such challenges, as well as students signalling their needs. Also, supervisors’ differing views about responsibility to support students appear to influence whether such support is provided or not. The findings here can contribute to discussions and policy regarding responsibilities and possibilities for offering adequate and realistic student support in relation to clinical placements under different conditions. Given the risk of negative consequences for students’ well-being and learning if their emotional challenges are ignored, it is of great importance to raise supervisors’ awareness and use of support strategies in this regard.

## Supplementary Information

Below is the link to the electronic supplementary material.

Supplementary Material 1

## Data Availability

No datasets were generated or analysed during the current study.
