# Supplementary material for: Protection, preparation or processing support for medical students’ emotional challenges in work-based learning—a grounded theory
Source: Adv Health Sci Educ Theory Pract. 2026 Feb 13;31(4):1239–58. doi: 10.1007/s10459-026-10512-4 (PMC13553703; doi:10.1007/s10459-026-10512-4)
Supplement: Supplementary file 1 [file 10459_2026_10512_MOESM1_ESM.pdf]

Guide for semi-structured interviews (translated from Swedish)

Probing to encourage participants to elaborate and give concrete examples were constantly used throughout the interviews.

Introductory Questions

How does supervision work in your department? What does it mean to be a supervisor in your department? (follow up with questions regarding concrete tasks)

How long have you been a supervisor? Have you participated in any supervision training?

What is important to you when you supervise students?

What do you think is the most positive aspects of being a supervisor?

What do you think characterises good supervision?

What role do you think the clinical training (work-based education) plays for students?

What do you consider especially important for students to take with them (learn) from their time with you/at this clinic/ward?

Perceptions of students' experiences of emotional challenges

Do you remember any situation during your clinical training that you found emotionally difficult? Did you receive support?

Have you noticed anything that students find emotionally difficult to handle?

What do you think students themselves experience as emotionally challenging?

Do you have experiences of situations in clinical practice where students have expressed emotional stress? What did you do?

What support do you think students need to manage emotionally difficult situations? What possibilities do you have to provide that support? (added after a few interviews: Who, according to you, is responsible to provide support?)

Do you think the study programme can prepare students for these kinds of emotionally challenging situations? How?

Supervision in emotionally difficult situations

Do you experience any challenges when it comes to being a supervisor? (If so, please elaborate)

Have you ever been in a situation where you felt you weren't good enough as a supervisor? Do you receive any support yourself when difficult situations arise in supervision?

What do you think about these emotionally challenging situations that students experience—how can they learn something from them?

Do you use specific strategies for supporting students if you notice they are distressed or seem emotionally affected? Can you give an example?

Have you discussed students' emotional challenging situations with others (colleagues / course coordinators)?
